# A Variable Precision Covering-Based Rough Set Model Based on Functions

**DOI:** 10.1155/2014/210129

**Published:** 2014-08-06

**Authors:** Yanqing Zhu, William Zhu

**Affiliations:** Lab of Granular Computing, Minnan Normal University, Zhangzhou 363000, China

## Abstract

Classical rough set theory is a technique of granular computing for handling the uncertainty, vagueness, and granularity in information systems. Covering-based rough sets are proposed to generalize this theory for dealing with covering data. By introducing a concept of misclassification rate functions, an extended variable precision covering-based rough set model is proposed in this paper. In addition, we define the *f*-lower and *f*-upper approximations in terms of neighborhoods in the extended model and study their properties. Particularly, two coverings with the same reductions are proved to generate the same *f*-lower and *f*-upper approximations. Finally, we discuss the relationships between the new model and some other variable precision rough set models.

## 1. Introduction

In the era of big data, it is difficult to obtain useful information in huge data. Many researchers have proposed lots of efficient means of dealing with the difficulty. As one of these efficient means, classical rough set theory based on equivalence relations is proposed by Pawlak [1, 2] in the early 1980s for handling the inexact, incomplete and uncertainty data. It is widely used for knowledge classification [3, 4] and rule learning [5–7]. On the one hand, owing to the restrictions of equivalence relations, many researchers have presented various extensions [8–13]. Especially, covering-based rough sets [14–20] are investigated as the extensions of classical rough set theory by extending partitions to coverings. On the other hand, in classical rough set model, the classification is fully correct and certain, and all conclusions are only applied to the set of objects. That largely limits its applications. Therefore, Ziarko [21] proposed the variable precision rough set model by introducing the measure of the relative degree of misclassification in classical rough set model. Moreover, some researchers have presented variable precision covering-based rough set models [22, 23] through extending variable precision rough sets.

In variable precision rough sets, the misclassification rate of equivalence classes of all elements in a universe is identical. Similarly, in variable precision covering-based rough sets, the misclassification rate of neighborhoods of all elements in a universe is identical too. However, in practical applications, since there are different understanding or demands about equivalence classes or neighborhoods of different elements, the misclassification rate usually varies. Hence, it is necessary to propose misclassification rate functions.

To address the above issue, we propose a variable precision covering-based rough set model based on functions by introducing misclassification rate functions in this paper. We present the concepts of the *f*-lower and *f*-upper approximations in terms of neighborhoods and investigate their properties.

The rest of this paper is arranged as follows.  Section 2 reviews some fundamental concepts of classical rough sets, covering-based rough sets and variable precision covering-based rough sets. In Section 3, we present the variable precision covering-based rough set model based on functions and investigate its properties. Meanwhile, we prove that two coverings with the same reductions generate the same *f*-lower and *f*-upper approximations. Moreover, the relationships between this model and some other variable precision rough set models are exhibited.  Section 4 concludes this paper and discusses some issues for further research.

## 2. Preliminaries

In this section, we present some fundamental concepts and existing results of classical rough sets, covering-based rough sets, and variable precision covering-based rough sets. Throughout this paper, the universe *U* is a non-empty finite set.

### 2.1. Classical Rough Sets

Let *U* be a universe and *R* an equivalence relation on *U*. *R* will generate a partition *U*/*R*. The elements in *U*/*R* are called equivalence classes. Let [*x*]_*R*_ denote an equivalence class which includes *x* ∈ *U*. For any *X*⊆*U*, we can describe *X* by the two sets,

(1) R_(X)={x∈U ∣ [x]R⊆X},R¯(X)={x∈U ∣ [x]R∩X≠∅}.

They are called the lower and upper approximations of *X* with respect to *R*, respectively.

### 2.2. Covering-Based Rough Sets

Covering-based rough sets are presented as the extension of classical rough sets by extending partitions to coverings on a universe.

**Definition 1 (see [17]).**

Let *U* be a universe of discourse and **C** a family of subsets of *U*. If none subsets in **C** is empty and ⋃**C** = *U*, then **C** is called a covering of *U*. The pair (*U*, **C**) is called a covering approximation space.

Neighborhoods are important concepts in covering-based rough sets.

**Definition 2 (see [24]).**

Let **C** be a covering of *U* and *x* ∈ *U*. *N*_**C**_(*x*) = ⋂{*K* ∈ **C**∣*x* ∈ *K*} is called the neighborhood of *x* with respect to **C**. When there is no confusion, we omit the subscript **C**.

**Definition 3 (see [24]).**

Let **C** be a covering of *U* and *x* ∈ *U*. For any *X*⊆*U*, the lower and upper approximations of *X* with respect to (*U*, **C**) are defined as follows, respectively:

 
$\underline{C}(X)=\{x\in U|N(x)\subseteq X\}$,
 
$\bar{C}(X)=\{x\in U|N(x)\cap X\neq \emptyset \}$.

Clearly, if **C** is a partition of  *U*, then covering-based rough sets degenerate into classical rough sets.

**Definition 4 (see [17]).**

Let **C** be a covering of *U* and *K* ∈ **C**. If *K* is a union of some sets in **C** − {*K*}, we say *K* is a reducible element of **C**; otherwise *K* is an irreducible element of **C**. The family of all irreducible elements of **C** is called the reduct of **C**, denoted as Reduct  (**C**).

### 2.3. Variable Precision Covering-Based Rough Sets

By introducing the parameter *β*  (0 ≤ *β* < 0.5) in classical rough sets, namely, some degrees of misclassification are allowed, Ziarko proposed variable precision rough sets.

Let *X* and *Y* be two non-empty subsets of  *U*. We say that *X* is included in *Y*, if every element of *X* is an element of *Y*. In this case, the inclusion relation is certain. That is to say, there does not exist the slightest misclassification between *X* and *Y*. However, slight misclassification is allowed in practical applications. Therefore, we present the majority inclusion relation which is a generalized definition of inclusion relation. Before the majority inclusion relation is presented, we introduce the measure of the relative degree of misclassification of one set with respect to others.

**Definition 5 (see [21]).**

Let *X* and *Y* be two subsets of a universe *U*. The measure *c*(*X*, *Y*) of the relative degree of misclassification of the set *X* with respect to set *Y* is defined as

(2) $$\begin{array}{c} c\left(X,Y\right)=\{\begin{array}{cc} 1-\frac{\left|X\cap Y\right|}{\left|X\right|}, & \left|X\right|\neq 0,\\ 0, & \left|X\right|=0, \end{array} \end{array}$$

where |*X*| denotes the cardinality of *X*.

**Definition 6 (see [21]).**

Let *X* and *Y* be two subsets of a universe *U*, and 0 ≤ *β* < 0.5. The majority inclusion relation is defined as

(3) $$\begin{array}{c} X\overset{\beta }{\subseteq }Y⟺c\left(X,Y\right)\leq \beta . \end{array}$$

By the definition, it is clear that *X*⊆*Y* if and only if *c*(*X*, *Y*) = 0.

**Proposition 7 (see [21]).**

If 0 ≤ *β*_1_ < *β*_2_ < 0.5, then $X\overset{\beta_{1}}{\subseteq }Y$ implies $X\overset{\beta_{2}}{\subseteq }Y$.

**Definition 8 (see [21]).**

Let *U* be a universe of discourse, *R* an equivalence relation, and 0 ≤ *β* < 0.5. For any *X*⊆*U*, the *β*-lower and *β*-upper approximations of *X* with respect to *R* are defined as follows, respectively:

 
${\underline{R}}_{\beta }(X)=\{x\in U|c({\left[x\right]}_{R},X)\leq \beta \}$,
 
${\bar{R}}_{\beta }(X)=\{x\in U|c({\left[x\right]}_{R},X)<1-\beta \}$.

When *β* = 0, it is easy to see that variable precision rough sets become classical rough sets. By extending partitions to coverings, variable precision rough sets are generalized to variable precision covering-based rough sets. We present the definition of variable precision covering-based rough sets as follows.

**Definition 9 (see [22]).**

Let **C** be a covering of *U* and 0 ≤ *β* < 0.5. For any *X*⊆*U*, the *β*-lower and *β*-upper approximations of *X* with respect to (*U*, **C**) are defined as follows, respectively:

 
${\underline{C}}_{\beta }(X)=\{x\in U|c(N(x),X)\leq \beta \}$,
 
${\bar{C}}_{\beta }(X)=\{x\in U|c(N(x),X)<1-\beta \}$.

Clearly, if *β* = 0, then variable precision covering-based rough sets are covering-based rough sets; if **C** is a partition of *U*, then variable precision covering-based rough sets are variable precision rough sets; if **C** is a partition of *U* and *β* = 0, then variable precision covering-based rough sets become classical rough sets.

## 3. Variable Precision Covering-Based Rough Sets Based on Functions

In variable precision rough sets, the misclassification rate of equivalence classes of all elements in a universe is identical. Similarly, in variable precision covering-based rough sets, the misclassification rate of neighborhoods of all elements in a universe is identical too. However, in practical applications, we have different understanding or demands about equivalence classes or neighborhoods of different elements. That means the misclassification rate usually varies. Therefore, we present variable precision covering-based rough sets based on functions by introducing a concept of misclassification rate functions.

Let **C** be a covering of *U* and *N*_**C**_(*x*) the neighborhood of *x*. It is obvious that ∪_*x*∈*U*_ *N*_**C**_(*x*) = *U*. That is, the collection of neighborhoods of all elements in *U* is still a covering of *U*. It is called neighborhood covering of *U* with respect to **C** and denoted by Cov(**C**) [25].

**Definition 10 .**

Let **C** be a covering of *U*. A function *f* defined from Cov(**C**) to [0,0.5) is called misclassification rate function.

**Definition 11 .**

Let **C** be a covering of *U* and *f* a misclassification rate function. For any *X*⊆*U*, the *f*-lower and *f*-upper approximations of *X* with respect to (*U*, **C**) are defined as follows, respectively:

 
${\underline{C}}_{f}(X)=\{x\in U|c(N(x),X)\leq f(N(x))\}$,
 
${\bar{C}}_{f}(X)=\{x\in U|c(N(x),X)<1-f(N(x))\}$.

**Example 12 .**

Let *U* = {*x*_1_, *x*_2_, *x*_3_, *x*_4_, *x*_5_, *x*_6_, *x*_7_, *x*_8_} and **C** = {*K*_1_, *K*_2_, *K*_3_}, where *K*_1_ = {*x*_1_, *x*_2_, *x*_3_, *x*_4_, *x*_5_}, *K*_2_ = {*x*_4_, *x*_5_, *x*_6_, *x*_7_} and *K*_3_ = {*x*_6_, *x*_8_}. Suppose that *f*(*N*(*x*)) = |*N*(*x*)|/2|*U*| and *X* = {*x*_2_, *x*_3_, *x*_4_, *x*_5_, *x*_6_}. Through the definition of neighborhoods, we have *N*(*x*_1_) = *N*(*x*_2_) = *N*(*x*_3_) = {*x*_1_, *x*_2_, *x*_3_, *x*_4_, *x*_5_}, *N*(*x*_4_) = *N*(*x*_5_) = {*x*_4_, *x*_5_}, *N*(*x*_6_) = {*x*_6_}, *N*(*x*_7_) = {*x*_4_, *x*_5_, *x*_6_, *x*_7_} and *N*(*x*_8_) = {*x*_6_, *x*_8_}. Therefore, *f*(*N*(*x*_1_)) = *f*(*N*(*x*_2_)) = *f*(*N*(*x*_3_)) = 5/16, *f*(*N*(*x*_4_)) = *f*(*N*(*x*_5_)) = *f*(*N*(*x*_8_)) = 2/16, *f*(*N*(*x*_6_)) = 1/16 and *f*(*N*(*x*_7_)) = 4/16. By Definition 5, *c*(*N*(*x*_*i*_), *X*) = 1/5  (*i* = 1,2, 3), *c*(*N*(*x*_*j*_), *X*) = 0  (*j* = 4,5, 6), *c*(*N*(*x*_7_), *X*) = 1/4 and *c*(*N*(*x*_8_), *X*) = 1/2. Hence ${\bar{C}}_{f}(X)=U$ and ${\underline{C}}_{f}(X)=\{x_{1},x_{2},x_{3},x_{4},x_{5},x_{6},x_{7}\}$. If *f* ≡ 0, then ${\bar{C}}_{f}(X)=U$ and $\underline{C}(X)=\{x_{4},x_{5},x_{6}\}$. If *f* ≡ *β* = 1/5, then ${\bar{C}}_{\beta }(X)=U$ and ${\underline{C}}_{\beta }(X)=\{x_{1},x_{2},x_{3},x_{4},x_{5},x_{6}\}$.

In practical applications, according to various needs, the different misclassification rate functions can be given by workers or researchers.

**Example 13 (continuation of Example 12).**

Suppose that *f*(*N*(*x*_*i*_)) = 0.1  (*i* = 1,…, 6) and *f*(*N*(*x*_7_)) = *f*(*N*(*x*_8_)) = 0.2. By Definition 11, ${\underline{C}}_{f}(X)=\{x_{4},x_{5},x_{6}\}$ and ${\bar{C}}_{f}(X)=U$.

Similar to classical rough sets or variable precision rough sets, we present the concepts of *f*-positive region Pos *c*_*f*_(*X*), *f*-boundary region Bn *c*_*f*_(*X*) and *f*-negative region Neg *c*_*f*_(*X*) of *X* with respect to (*U*, **C**) by the following definition.

**Definition 14 .**

Let *U* be a universe of discourse and *f* a misclassification rate function. For any *X*⊆*U*, the *f*-positive region Pos *c*_*f*_(*X*), *f*-boundary region Bn *c*_*f*_(*X*) and *f*-negative region Neg *c*_*f*_(*X*) of *X* with respect to (*U*, **C**) are defined as follows, respectively:

1. ${\text{Pos}\;c}_{f}(X)={\underline{C}}_{f}(X)$,
2. ${\text{Bn}\;c}_{f}(X)={\bar{C}}_{f}(X)-{\underline{C}}_{f}(X)$,
3. ${\text{Neg}\;c}_{f}(X)=U-{\bar{C}}_{f}(X)$.

**Proposition 15 .**

Let *U* be a universe of discourse. For any *X*⊆*U*, the following conclusions are true,

1. Neg *c*_*f*_(*X*) = {*x* ∈ *U*∣*c*(*N*(*x*), *X*) ≥ 1 − *f*(*N*(*x*))},
2. Bn *c*_*f*_(*X*) = {*x* ∈ *U*∣*f*(*N*(*x*)) < *c*(*N*(*x*), *X*) < 1 − *f*(*N*(*x*))},
3. Pos *c*_*f*_(−*X*) = Neg *c*_*f*_(*X*).

**Proof**

(1) ${\text{Neg}\;c}_{f}\left(X\right)=U-{\bar{C}}_{f}\left(X\right)=U-\left\{x\in U|c\left(N\left(x\right),X\right)<1-f\left(N\left(x\right)\right)\right\}$  =  {*x* ∈ *U*∣*c*(*N*(*x*), *X*) ≥ 1 − *f*(*N*(*x*))}.

(2) ${\text{Bn}\;c}_{f}\left(X\right)={\bar{C}}_{f}\left(X\right)-{\underline{C}}_{f}\left(X\right)=\left\{x\in U|c\left(N\left(x\right),X\right)<1-f\left(N\left(x\right)\right)\right\}-\left\{x\in U|c\left(N\left(x\right),X\right)\leq f\left(N\left(x\right)\right)\right\}$  =  {*x* ∈ *U*∣*f*(*N*(*x*)) < *c*(*N*(*x*), *X*)<1 − *f*(*N*(*x*))}.

(3) For any *x* ∈ *U*, *c*(*N*(*x*), −*X*) = 1 − *c*(*N*(*x*), *X*). ${\text{Pos}\;c}_{f}\left(-X\right)={\underline{C}}_{f}(-X)=\left\{x\in U|c\left(N\left(x\right),-X\right)\leq f\left(N\left(x\right)\right)\right\}$  =  {*x* ∈ *U*∣1 − *c*(*N*(*x*), *X*) ≤ *f*(*N*(*x*))}  =  {*x* ∈ *U*∣*c*(*N*(*x*), *X*) ≥ 1 − *f*(*N*(*x*))} = Neg *c*_*f*_(*X*).

In the following definition, we present the *f*-accuracy and *f*-roughness, which are important numerical characteristics of this type of rough sets.

**Definition 16 .**

Let **C** be a covering of *U*. For any *X*⊆*U*, the *f*-accuracy and *f*-roughness are defined as follows, respectively:

 
$\lambda_{f}\left(X\right)=\left|{\underline{C}}_{f}\left(X\right)\right|/\left|{\bar{C}}_{f}\left(X\right)\right|$,
 
*ρ*_*f*_(*X*) = 1 − *λ*_*f*_(*X*).

### 3.1. Basic Properties

In this subsection, we present the properties and some significant results concerning the new model.

**Theorem 17 .**

Let **C** be a covering of  *U* and *f* a misclassification rate function. For all *X*, *Y*⊆*U*, the following conclusions are true,

1. ${\underline{C}}_{f}(X)\subseteq {\bar{C}}_{f}(X)$,
2. ${\underline{C}}_{f}(\emptyset )={\bar{C}}_{f}(\emptyset )=\emptyset$, ${\underline{C}}_{f}(U)={\bar{C}}_{f}(U)=U$,
3. If *X*⊆*Y*, then ${\underline{C}}_{f}(X)\subseteq {\underline{C}}_{f}(Y)$, ${\bar{C}}_{f}(X)\subseteq {\bar{C}}_{f}(Y)$,
4. ${\underline{C}}_{f}(X)\cup {\underline{C}}_{f}(Y)\subseteq {\underline{C}}_{f}(X\cup Y)$,
5. ${\bar{C}}_{f}(X)\cup {\bar{C}}_{f}(Y)\subseteq {\bar{C}}_{f}(X\cup Y)$,
6. ${\underline{C}}_{f}(X\cap Y)\subseteq {\underline{C}}_{f}(X)\cap {\underline{C}}_{f}(Y)$,
7. ${\bar{C}}_{f}(X\cap Y)\subseteq {\bar{C}}_{f}(X)\cap {\bar{C}}_{f}(Y)$,
8. ${\underline{C}}_{f}(-X)=-{\bar{C}}_{f}(X)$, ${\bar{C}}_{f}(-X)=-{\underline{C}}_{f}(X)$.

**Proof**

(1) For any *x* ∈ *U*, *f*(*N*(*x*)) < 0.5 < 1 − *f*(*N*(*x*)). By Definition 11, it is clear that ${\underline{C}}_{f}(X)\subseteq {\bar{C}}_{f}(X)$.

(2) For any *x* ∈ *U*, since *c*(*N*(*x*), *∅*) = 1, it follows that ${\underline{C}}_{f}(\emptyset )={\bar{C}}_{f}(\emptyset )=\emptyset$. Similarly, since *c*(*N*(*x*), *U*) = 0, it follows that ${\underline{C}}_{f}(U)={\bar{C}}_{f}(U)=U$.

(3) If *X*⊆*Y*, then *c*(*N*(*x*), *Y*) ≤ *c*(*N*(*x*), *X*). Hence, ${\underline{C}}_{f}(X)\subseteq {\underline{C}}_{f}(Y)$ and ${\bar{C}}_{f}(X)\subseteq {\bar{C}}_{f}(Y)$.

(4) For all *X*, *Y*⊆*U*, since *c*(*N*(*x*), *X* ∪ *Y*) ≤ *c*(*N*(*x*), *X*) and *c*(*N*(*x*), *X* ∪ *Y*) ≤ *c*(*N*(*x*), *Y*), it follows that ${\underline{C}}_{f}(X)\cup {\underline{C}}_{f}(Y)\subseteq {\underline{C}}_{f}(X\cup Y)$.

(5) Similar to the proof of (4).

(6) For all *X*, *Y*⊆*U*, since *c*(*N*(*x*), *X*) ≤ *c*(*N*(*x*), *X*∩*Y*) and *c*(*N*(*x*), *Y*) ≤ *c*(*N*(*x*), *X*∩*Y*), it follows that ${\underline{C}}_{f}(X\cap Y)\subseteq {\underline{C}}_{f}(X)\cap {\underline{C}}_{f}(Y)$.

(7) Similar to the proof of (6).

(8) For any *x* ∈ *U*, *c*(*N*(*x*), −*X*) = 1 − *c*(*N*(*x*), *X*). Hence, ${\underline{C}}_{f}(-X)=-{\bar{C}}_{f}(X)$. It is easy to see that ${\bar{C}}_{f}(-X)=-{\underline{C}}_{f}(X)$.

**Theorem 18 .**

Let **C** be a covering of *U* and *f*, *g* two misclassification rate functions. Suppose that *f*(*N*(*x*)) ≤ *g*(*N*(*x*)) for any *x* ∈ *U*. For every *X*⊆*U*, the following relationships are true,

1. ${\underline{C}}_{f}(X)\subseteq {\underline{C}}_{g}(X)$,
2. ${\bar{C}}_{g}(X)\subseteq {\bar{C}}_{f}(X)$.

**Proof**

According to Proposition 7 and Definition 11, (1) and (2) are straightforward.

**Corollary 19 .**

Let **C** be a covering of *U* and *f* a misclassification rate function. Denote *α*_1_ = min⁡⁡{*f*(*N*(*x*))∣*N*(*x*) ∈ Cov (**C**)} and *α*_2_ = max⁡⁡{*f*(*N*(*x*))∣*N*(*x*) ∈ Cov (**C**)}. For any *X*⊆*U*, then

1. ${\underline{C}}_{\alpha_{1}}(X)\subseteq {\underline{C}}_{f}(X)\subseteq {\underline{C}}_{\alpha_{2}}(X)$,
2. ${\bar{C}}_{\alpha_{2}}(X)\subseteq {\bar{C}}_{f}(X)\subseteq {\bar{C}}_{\alpha_{1}}(X)$.

**Theorem 20 .**

Let **C**, **D** be two different coverings of *U* and *f* a misclassification rate function. If *Reduct*  (**C**) = *Reduct*  (**D**), then ${\underline{C}}_{f}(X)={\underline{D}}_{f}(X)$ and ${\bar{C}}_{f}(X)={\bar{D}}_{f}(X)$ for any *X*⊆*U*.

**Proof**

From Definition 11, we need to prove Cov(**C**) = Cov(**D**). Suppose that *K*_1_ is a reducible element of **C**. For any *x* ∈ *U*, *x* ∈ *K*_1_ or *x* ∉ *K*_1_. If *x* ∉ *K*_1_, it is straightforward that *N*_**C**_(*x*) = *N*_**C**−{*K*_1_}_(*x*). Otherwise, there exists *K*′ ⊂ *K*_1_ such that *x* ∈ *K*′ and *K*′ is an irreducible element of **C**. Thus, *N*_**C**_(*x*) = ⋂_*x*∈*K*∈**C**_ *K* = (⋂_*x*∈*K*∈**C**−{*K*′,*K*_1_}_ *K*)⋂*K*′⋂*K*_1_ = (⋂_*x*∈*K*∈**C**−{*K*′,*K*_1_}_ *K*)⋂*K*′ = *N*_**C**−{*K*_1_}_(*x*). Hence *N*_**C**_(*x*) = *N*_**C**−{*K*}_(*x*) for any reducible element *K* ∈ **C**. According to this method, it is easy to see that *N*_**C**_(*x*) = *N*_Reduct  (**C**)_(*x*). From the arbitrariness of *x*, Cov(**C**) = Cov(Reduct  (**C**)). Since Reduct  (**C**) = Reduct  (**D**), it follows that Cov(**C**) = Cov(**D**). This completes the proof.

**Example 21 .**

Let *U* = {*x*_1_, *x*_2_, *x*_3_, *x*_4_, *x*_5_, *x*_6_, *x*_7_, *x*_8_}. **C** = {*K*_1_, *K*_2_, *K*_3_, *K*_4_}, where *K*_1_ = {*x*_1_, *x*_2_, *x*_3_}, *K*_2_ = {*x*_3_, *x*_4_, *x*_5_}, *K*_3_ = {*x*_4_, *x*_5_, *x*_6_, *x*_7_} and *K*_4_ = {*x*_6_, *x*_8_}. **D** = {*K*_1_, *K*_2_, *K*_3_, *K*_4_}, where *K*_1_ = {*x*_1_, *x*_2_, *x*_3_, *x*_4_, *x*_5_}, *K*_2_ = {*x*_4_, *x*_5_, *x*_6_, *x*_7_}, *K*_3_ = {*x*_6_, *x*_8_} and *K*_4_ = {*x*_4_, *x*_5_, *x*_6_, *x*_7_, *x*_8_}. Clearly, Reduct  (**C**) = Reduct  (**D**). Suppose *X* = {*x*_2_, *x*_3_, *x*_4_, *x*_5_, *x*_6_}. Hence ${\bar{C}}_{f}(X)={\bar{D}}_{f}(X)=U$ and ${\underline{C}}_{f}(X)={\underline{D}}_{f}(X)=\{x_{1},x_{2},x_{3},x_{4},x_{5},x_{6},x_{7}\}$.

However, the converse proposition of Theorem 20 is not true. The following example will illustrate that.

**Example 22 .**

Let **C** be the covering in Example 21. **D** = {*K*_1_, *K*_2_, *K*_3_, *K*_4_}, where *K*_1_ = {*x*_1_, *x*_2_, *x*_3_, *x*_4_, *x*_5_}, *K*_2_ = {*x*_4_, *x*_5_, *x*_6_, *x*_7_}, *K*_3_ = {*x*_6_, *x*_8_} and *K*_4_ = {*x*_1_, *x*_2_, *x*_3_, *x*_4_, *x*_5_, *x*_6_}. Suppose that *X* = {*x*_2_, *x*_3_, *x*_4_, *x*_5_, *x*_6_}. It is easy to know that ${\underline{C}}_{f}(X)={\underline{D}}_{f}(X)$ and ${\bar{C}}_{f}(X)={\bar{D}}_{f}(X)$. But Reduct  (**C**) ≠ Reduct  (**D**).

### 3.2. Relationships among Five Types of Rough Sets

**Figure 1:**
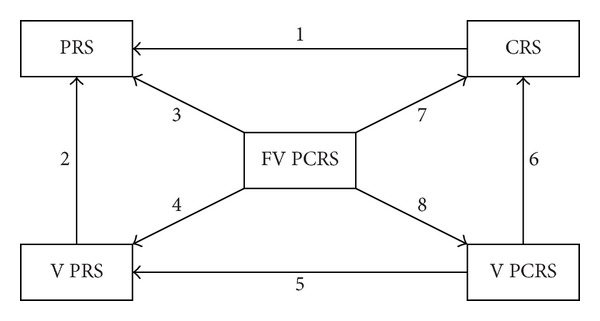
Relationships among five types of rough sets.

In this subsection, we will use a figure (Figure 1) to explain the relationships among five types of rough sets: classical rough sets (PRS), covering-based rough sets (CRS), variable precision rough sets (VPRS), variable precision covering-based rough sets (VPCRS) and variable precision covering-based rough sets based on functions (FVPCRS). In order to easily understand Figure 1, we give some notes below. Let *f* be a misclassification rate function and *β* a constant, where 0 ≤ *β* < 0.5.

“1”: Cov(**C**) is a partition of *U*;
“2”: *β* = 0;
“3”: Cov(**C**) is a partition of *U* and *f* ≡ 0;
“4”: Cov(**C**) is a partition of *U* and *f* ≡ *β*;
“5”: Cov(**C**) is a partition of *U*;
“6”: *β* = 0;
“7”: *f* ≡ 0;
“8”: *f* ≡ *β*.

## 4. Conclusions and Future Work

In this paper, we proposed the variable precision covering-based rough set model based on functions as a generalization of a variable precision covering-based rough set model and studied its properties. Through the concept of reductions, we obtained that two coverings with the same reductions generate the same *f*-lower and *f*-upper approximations. Moreover, we exhibited the relationships between this model and some other variable precision rough set models. In future work, we will seek more specific misclassification rate functions for dealing with more types of data.
